# Association between *ANKK1* (rs1800497) and *LTA* (rs909253) Genetic Variants and Risk of Schizophrenia

**DOI:** 10.1155/2015/821827

**Published:** 2015-05-31

**Authors:** Arwa H. Arab, Nasser A. Elhawary

**Affiliations:** ^1^Department of Psychology, Faculty of Arts and Humanities, King Abdulaziz University, P.O. Box 80200, Jeddah 21589, Saudi Arabia; ^2^Department of Medical Genetics, Faculty of Medicine, Umm Al-Qura University, P.O. Box 57543, Mecca 21955, Saudi Arabia; ^3^Department of Molecular Genetics, Faculty of Medicine, Ain Shams University, Cairo 11566, Egypt

## Abstract

Limited research has assessed associations between schizophrenia and genetic variants of the ankyrin repeat and kinase domain containing 1 (*ANKK1*) and lymphotoxin-alpha (*LTA*) genes among individuals of Middle Eastern ancestry. Here we present the first association study investigating the *ANKK1* rs1800497 (T>C) and *LTA* rs909253 (A>G) single-nucleotide polymorphisms in an Egyptian population. Among 120 patients with DSM-IV and PANSS (Positive and Negative Syndrome Scale) assessments of schizophrenia and 100 healthy controls, we determined the genotypes for the polymorphisms using endonuclease digestion of amplified genomic DNA. Results confirmed previous findings from different ethnic populations, in that the rs1800497 and rs909253 polymorphisms were both associated with risk of schizophrenia. Differences between the genotypes of cases and controls were strongly significant (*P* = 0.0005 for rs1800497 and *P* = 0.001 for rs909253). The relative risk to schizophrenia was 1.2 (*P* = 0.01) for the C allele and 0.8 (*P* = 0.04) for the G allele. The CC, GG, and combined CC/AA genotypes were all more frequent in cases than in controls. These results support an association between *ANKK1* and *LTA* genetic markers and vulnerability to schizophrenia and show the potential influence of just one copy of the mutant C or G allele in the Egyptian population.

## 1. Introduction

Schizophrenia is a highly heritable disorder characterized by psychotic symptoms, alterations of thought, volition, and abnormal perceptions (i.e., hallucinations) and beliefs (i.e., delusions). With a worldwide lifetime prevalence of 0.5–2% [1], it is noteworthy that no society or culture in the world is free from schizophrenia. Although few data are available on incidence in developing countries, early assumptions about consistently lower rates outside the western industrialized countries have not been confirmed by investigations in Asian countries [2, 3].

Supporting evidence has linked the high heritability of schizophrenia to a combination of relatively common alleles of small effect and to a few rare alleles with relatively large effects [4]. Thirteen genetic SNPs contributing to schizophrenia have been identified in recent years through genome-wide association studies [5–8] and surpassing a genome-wide significance threshold [9] of *P* < 5 × 10^−8^. These studies have identified 10 genes (i.e.,* CSF2RA*,* HIST1H2BJ*,* NOTCH4*,* NRGN*,* SHOX*,* SMARCA2*,* TCF4*,* ZNF804A*,* PRSS16*, and* PGBD1*) [10–14], but they confer relatively small increments in risk and explain only a small proportion of heritability [14, 15].

Some of the most investigated genes in studies of susceptibility to schizophrenia are those that encode proteins of the dopaminergic system. Evidence suggests a role of central dopamine pathways in the pathophysiology of the disorder [16], as drugs that reduce dopamine levels diminish psychotic symptoms and drugs that increase dopamine levels exacerbate these symptoms [17].

The rs1800497 single-nucleotide polymorphism (SNP) has been identified within exon 8 of the ankyrin repeat and kinase domain containing 1 (*ANKK1*) gene (MIM 608774), just 10 kb away from* the dopamine receptor D2* (*DRD2*) gene (located at 11q23.2, MIM 126450) in the 3′ untranslated region. This polymorphism, which leads to a substitution of glutamic acid for a basic lysine (E713K) that may alter substrate-binding specificity [18], likely modulates the function and expression of* DRD2* due to its close proximity [19]. Despite the fact that the rs1800497 SNP is localized to the* ANKK1* gene, it seems to be in linkage disequilibrium with several* DRD2* genetic variants, which could potentially explain a dopaminergic role in the etiopathogenesis of schizophrenia [20].

The rs1800497 pathogenic variant has been previously described to associate with a reduction in DRD2 receptor density in the brain [21] and thus may be a risk factor for neuropsychiatric disorders. Many studies have also supported the association between the rs1800497 SNP and drug dependence [21–23], although others have not found such an association [24].

The lymphotoxin-alpha (*LTA*; MIM 153440) gene and tumor necrosis factor-alpha (*TNF-α*; MIM 191160) gene, located 13 kb apart on chromosome 6p21, are cytokines functioning as the principal mediators of the immune response. The* LTA* gene is known to play a central role in neurodevelopment, synaptic plasticity, and the response to neural injury [25]. Repeatedly associated with various brain activities and having immunologic, neurochemical, neuroendocrine, and behavioral effects, the* LTA* gene has also been associated with symptoms of schizophrenia [26]. In contrast to studies of TNF-*α*, few studies have evaluated the association between* LTA* gene polymorphisms and susceptibility to schizophrenia [27–29].

Despite a large amount of available genetic information on schizophrenia, most of the reports are from western countries. Only 18 articles have focused on this psychotic disorder in Middle Eastern populations; two of them are from Egypt, describing likely associations between the disorder and the methylenetetrahydrofolate reductase [30] and brain-derived neurotrophic factor [31] genes. These documentations, taken together with the strongest support for the involvement of dopamine as antipsychotic drugs, and the neurodevelopmental evidence of lymphotoxin as a cytokine in signaling between cells during immune response, have highlighted the* ANKK1* and the* LTA* as promising schizophrenia susceptibility genes.

In the present case-control study, we focus on polymorphisms of* ANKK1* and* LTA* as possible risk factors for schizophrenia. Specifically, we hypothesized that the C allele within the rs1800497 SNP of* ANKK1* and the G allele within the rs909253 SNP of* LTA* may affect vulnerability to schizophrenia among Egyptian cases. To the best of our knowledge, this is the first study to evaluate possible associations between* ANKK1* and* LTA* genetic variants and schizophrenia in this population.

## 2. Subjects and Methods

### 2.1. Characteristics of the Study Population

The study was conducted among Egyptian patients diagnosed with schizophrenia and healthy controls. Patients aged 20–55 years who presented at the Outpatient Psychiatry Clinics, Psychiatry Department, Ain Shams University, Cairo, over a three-month period were considered for inclusion. The mean age was 27.6 years at onset and 32.6 years at examination. The gender distribution of the patients was 1 : 1.5 (women: men). The mean duration of the schizophrenia was 9.8 years (standard deviation (SD), ±4.7 years). The patients were recruited from both rural and urban areas with an equal ratio and were of Egyptian origin.

The proportion of patients with insidious-onset schizophrenia (62%) was higher than the proportion with acute-onset disease (38%). Use of antipsychotics for treatment of behavioral and psychological symptoms of schizophrenia was frequent among patients. Among users, there were significantly more used typical medications (70%) than atypical (30%) or typical-atypical ones (40%).

Diagnosis of schizophrenia was confirmed according to the criteria of the* Diagnostic and Statistical Manual of Mental Disorders*, Fourth Edition (DSM-IV), based on individual interviews, clinical observation, medical records (hospital and outpatient clinic case notes), and family information (American Psychiatric Associations, 1994). Moreover, the patients were subjected to the Positive and Negative Syndrome Scale (PANSS) [32] assessment using a 45- to 50-minute interview to measure the severity of a patient's schizophrenia. “Positive symptoms” referred to an excess or distortion of normal functions (e.g., hallucinations and delusions) and the “negative symptoms” described a diminution or loss of normal functions. In the interview, 30 symptoms (7 positive symptoms, 7 negative symptoms, and 16 symptoms of general psychopathology) were each rated from 1 to 7. The total PANSS score (with a maximum of 210) could not be lower than 30 for a schizophrenic patient. The mean PANSS scores were 92.8 (SD, ±13.56) for the total score, 23.1 (SD, ±7.91) for positive psychotic symptoms, 22.7 (SD, ±4.16) for negative psychotic symptoms, and 47.8 (SD, ±7.79) for general psychopathology.

Those individuals with a history of traumatic brain injury, neurologic disorders, mental retardation, epilepsy, or substance addiction (except for nicotine) were excluded. The control group consisted of healthy volunteers who did not meet any of the exclusion criteria and who were free from present, past, and a family history (first-degree relatives) of psychiatric illness.

This study was approved by the local Biomedical Ethics Committee at Faculty of Medicine, Umm Al-Qura University, and by the Psychiatry Department, Faculty of Medicine, Ain Shams University, Cairo. Informed and written consent was obtained from each participant prior to enrollment in the study.

### 2.2. DNA Analysis

High molecular weight DNA samples were extracted from saliva using the Oragene.DNA-OG-500 kit (DNA Genotek Inc., Ottawa, ON, Canada). The full saliva sample should be collected within 30 minutes and the Oragene tube should be capped immediately. The saliva was incubated with the lysis buffer at 50°C to release the DNA that was then precipitated by ethanol and dissolved in Tris-EDTA buffer, as recommended by the manufacturer.

### 2.3. Genotyping of* ANKK1* rs1800497 and* LTA* rs909253

The* ANKK1* rs1800497 (T>C) and* LTA* rs909253 (A>G) SNPs were genotyped using* TaqI* and* NcoI* restriction endonucleases. The rs1800497 locus was amplified using the forward 5′-CCG TCG ACG GCT GGC CAA GTT TCT A-3′ and reverse 5′-CCG TCG ACC CTT CCT GAG TGT CAT CA-3′ primers [33], whereas the rs909253 locus was amplified using the 5′-CCG TGC TTC GTG CTT TGG ACT A-3′ and 5′-AGA GCT GGT GGG GAC ATG TCG-3′ primers [34, 35] at an annealing temperature of 58°C for both variants. Following amplification, 5 *μ*L of PCR amplicon was incubated overnight with 5 U of* TaqI* and* NcoI* at 65° and 37°C, respectively, as recommended by the manufacturer (Fermantas, GmbH, Germany). The fragments were separated on a 3% MetaPhor agarose gel (BMA, Rockland, ME) using ethidium bromide staining and viewed under a UV transilluminator (G-Box, Syngene, Frederick, MD).

The amplicon with the T allele remained uncut (310 bp), but that with the C allele was cleaved into 180 and 130 bp fragments. The* LTA* +252G allele was identified by 545 and 196 bp fragments, and the* LTA* +252A allele by a single 741 bp fragment. A positive control was used for each variant. Each sample was run in duplicate. The genotypes of all DNA samples were reassessed twice to confirm the results and ensure reproducibility. Some suspected genotypes were validated by purifying the PCR products using an automated Agencourt AMPure XP kit (Beckman Coulter Inc., Riyadh, Saudi Arabia) and genotyping using a Genetic Analyzer 3500 (ABI, Life Technologies, Jeddah, Saudi Arabia).

### 2.4. Data Analysis

Hardy-Weinberg equilibria of the genetic variants rs1800497 and rs909253 were assessed using online software (http://www.oege.org/software/hwe-mr-calc.shtml). Student's *t*-test was used to compare sociodemographic and clinical characteristics. The Mantel-Haenszel* chi*-square (*χ* ^2^) test for linear association was used to assess the contributions of the genotypes and alleles of rs1800497 and rs909253 and other independent risk factors to the study outcomes (http://www.socscistatistics.com/tests/chisquare/Default.aspx). All *P* values were two-tailed, and a probability < 0.05 was considered statistically significant. The odds ratios and relative risks associated with the C allele (rs1800497) and the G allele (rs909253), their standard errors, 95% confidence intervals (CIs), and* z*-scores were calculated using MedCalc software for Windows, version 12.3.0.0 (Mariakerke, Belgium).

We used G*∗*Power software, version 3.1.5 (Universität Kiel, Germany; http://www.psycho.uni-duesseldorf.de/abteilungen/aap/gpower3/download-and-register/) to perform* a priori* power analysis to estimate sufficient sample sizes to achieve adequate power for *z*-testing of two independent proportions.* A priori* sample-size estimations were performed using known information on the common allele frequencies in schizophrenia patients, a criterion probability of *α* = 0.05, and a power sensitivity of 80%. The prevalence of schizophrenia in the studied population was assumed to be 50%, with a case-control ratio of 1.

## 3. Results

For the study, 120 eligible individuals with schizophrenia (72 men and 48 women) and 100 healthy nonpsychotic controls were enrolled.  Table 1 shows selected sociodemographic and clinical characteristics. Thirty additional eligible patients with schizophrenia did not enroll because they refused to be clinically investigated, they could not be traced, or their clinical profiles were incomplete.

### 3.1. Hardy-Weinberg Equilibria of* ANKK1* and* LTA* Variants

None of the rs1800497 or rs909253 SNPs showed deviation from Hardy-Weinberg equilibria in the control population (*χ* ^2^ = 3.6; *P* = 0.06, and *χ* ^2^ = 0.5; *P* = 0.5, resp.).

### 3.2. Association with the rs1800497 (T>C) Variant

As shown in Table 2(a), the mutant C allele was significantly more frequent in schizophrenia cases than in controls (82% versus 72%). The odds ratio of the C allele outcome was 1.8 (95% CI, 1.2–2.9; *z* = 2.9; *P* = 0.009); consequently, the relative risk of the C allele outcome was 1.2 (95% CI, 1.0–1.3; *z* = 2.6; *P* = 0.01). As shown in Table 2(b), genotypic analysis of the rs1800497 polymorphism showed the absence of TT carriers among schizophrenia cases but showed a 12% prevalence among controls. The CC genotype was more frequent in schizophrenia cases than in controls (64.2% versus 55%). The difference between the genotypes of schizophrenia cases and controls containing the C allele was highly significant (*χ* ^2^ = 15.3; *P* = 0.0005).

### 3.3. Association with the rs909253 (A>G) Variant

The odds ratio of the mutant G allele outcome was 0.7 (95% CI, 0.5–1.0; *z* = 2.1; *P* = 0.04), and the relative risk of the G allele outcome was 0.8 (95% CI, 0.6–1.0; *z* = 2.1; *P* = 0.04). The wild-type A allele was more frequent than the mutant G allele in both cases (67.5% versus 3.5%) and controls (58% versus 42%) (Table 2(a)). The mutant GG genotype was more frequent in schizophrenia cases (18.3%) than in controls (16%). The difference between the genotypes of schizophrenia cases and controls containing the G allele was significant (*χ* ^2^ = 13.7; *P* = 0.001) (Table 2(b)).

### 3.4. Combined Genotypes of* ANKK1/LTA* Variants

Table 3 shows the combined genotype effect of the two SNPs, rs1800497 (T>C) and rs909253 (A>G), on the risk for schizophrenia. Among the nine possible genotypes, we found only six combined genotypes in schizophrenia cases; the CC/AA was the most frequent (40%), followed by CC/AG (15%). Among the controls, the TT/AA and CC/GG genotypes were the least prevalent. The nine combined genotypes differed significantly between cases and controls (*χ* ^2^ = 85.8; df = 8; *P* = 0). Regression analysis showed no significant differences between the cases and controls (*r* = 0.56).

## 4. Discussion

We have presented the first association study investigating the* ANKK1* rs1800497 (T>C) and* LTA* rs909253 (A>G) SNPs in an Egyptian sample of patients with schizophrenia. Overall, our results provide strong evidence of associations between these two SNPs and risk of schizophrenia in this population. Moreover, the frequencies of the C allele (rs1800497) and the G allele (rs909253) were found to potentially affect the risk of schizophrenia when compared with those of wild-type alleles (*P* = 0.009 and *P* = 0.04, resp.).

In addition, we found no deviation from Hardy-Weinberg equilibria for either SNP in our controls. Most previous observations in* ANKK1* are consistent with this equilibrium, with the exception of two previous reports showing significant deviations with the rs1800497 SNP [33, 36].

Data on linkages or associations between the* ANKK1* locus and schizophrenia vary among different ethnicities. A meta-analysis recently reported that the rs1800497 SNP is cumulatively associated with schizophrenia among East Asians (*P* = 0.007) but not among Caucasians (*P* = 0.245) or among Indians and Sri Lankans (*P* = 0.329) [37]. Our study in the Egyptian population, as well as studies among American (non-Hispanic white) and French (Caucasian) populations, has further revealed a highly significant association between the C allele of the rs1800497 SNP and schizophrenia [21, 38] (Table 4). A study among Brazilian-Latino patients also suggested borderline significance (*P* = 0.06).

In contrast, studies in Russian, Indian, Chinese, Iranian, and German populations have reported insignificant differences between schizophrenia cases and controls [36, 39–43]. The relatively small sample sizes of the Indian and Iranian populations might have affected these results. An additional study reported a near founder effect of the* ANKK1* rs1800497 polymorphism among the Turkish population with schizophrenia (*n* = 99) of 97.2% for the T allele and 2.8% for the C allele, reporting no significant differences in C and T allele frequencies between cases and controls [44].

It has been challenging to find a plausible explanation for these contradictions based on gene flow or genetic drift within ethnic populations. Yet, we can justify them based on genetic heterogeneity between continental populations or based on lifestyle, socioeconomic, and other environmental factors that can positively or negatively affect morbidity and mortality among East Asian, Caucasian, Latina, Indian, or Egyptian patients with severe phenotypes [45].

Despite the potential role of neurotransmitters, such as dopamine, in the risk of schizophrenia, and in the development of newer antipsychotics based on these hypotheses until several decades ago, more cytokines have still shared a vulnerability for their influence on schizophrenia. The immunomodulatory functions of* LTA* are well known, particularly in cellular defense against viral infection and phagocyte-dependent inflammation [34]. LTA has been found to be protective against excitotoxicity. Expression of the* LTA* mRNA is observed in areas of white and gray matter, which are highly sensitive to excitotoxicity [46]. Alterations in myelin and white matter may cause aberration of neurotransmission resulting in a disharmonious information processing between subcortical and cortical networks that maybe associated with psychosis exacerbation [46]. In particular,* LTA* has been shown to be more effective than TNF-*α* in protecting neurons against glutamate and* N*-methyl-*D*-aspartate (NMDA) toxicity [47].

Our finding of a statistically significant association between the* LTA* rs909253 polymorphism and schizophrenia supports a previous hypothesis of pathophysiology of* LTA* gene expression. Our result is consistent with a report showing an association between this particular SNP and schizophrenia in the Korean population (odds ratio = 1.76; 95% CI = 1.27–2.45; *P* = 0.0007) [27]. However, in contrast to our study, the mutant G allele of rs909253 was prevalent and overexpressed in the Korean population.

Another study has found a genetic variant in the noncoding sequence of the first exon of* LTA* (rs1800683) to be significantly associated with cognitive functioning within schizophrenia patients (*P* < 0.008) [48]. Associations have also been found for other genetic loci linked to the short arm of chromosome 6. These include the rs111602326 and rs113663679 loci within the* ARVD8* gene (MIM 607450) [49], the* HLA-DRB1* gene (MIM 142857) [50], and the rs113785696 and rs111341380 loci associated with schizophrenia type 3 (MIM 600511) [51, 52].

In our study, combined genotyping of the* ANKK1*/*LTA* variants revealed no significant differences between schizophrenia cases and controls (*r* = 0.56). However, it is interesting that in cases, combined genotypes containing the mutant CC genotype were more frequent than combined genotypes containing the TT genotype. In contrast, combined genotypes containing the mutant GG genotype were less frequent than those containing the wild-type AA genotype.

Although schizophrenia is a major public health concern causing extensive suffering and requiring costly treatment and care, only a few reports provide evidence on genetic-environmental interactions that affect the risk of schizophrenia. Despite the significant contribution of genetic factors to the expression of syndromes, like schizophrenia, these syndromes may be a heterogeneous collection of genetic and nongenetic illnesses. To limit the potential for genetic heterogeneity, we performed our study on an Egyptian sample population of historically relative homogeneity. The random selection of our patients with schizophrenia gave rise to an equal ratio of urban and rural Egyptians. This enabled us to exclude the influence of social contacts, such as family visits, social visits, and planned social activities, which have been observed as less common in urban than rural environments [53]. Some trials have reported that rural living is associated with a greater frequency of social contacts among patients with schizophrenia [53], but the risk of the disease in the most urban environments is estimated to be more frequent (2.37 times) compared to that in the most rural environments [54].

Conflicting results in pinning down a genetic association for schizophrenia are not uncommon, quite the contrary, as poor replication can arise from several different factors. The present study had a few limitations. First, some other populations have been of admixed ethnicities and/or are expressed in small sample sizes, which would lessen the strength of the overall results to be consistent with our Egyptian populations. Also, our* post hoc* statistical analyses for the* ANKK1* rs1800497 and* LTA* rs909253 SNPs revealed powers of 49% and 33%, respectively, among our 220 participants. Usually smaller sample sizes lead to lower* post hoc* power of detection, coinciding with increased rates of both false-positives and false-negatives and subsequent difficulty replicating or contradicting previous findings. Overcoming these limitations can be difficult, but sometimes meta-analysis offers some viable options as it permits easily surveying a wide set of subjects, thereby enhancing* post hoc* power and allowing for a more broadly based analysis of previously available data. Recruiting more participants (i.e., 466 for rs1800497 and 730 for rs909253) would have been needed to reach a power of 80%. But recruiting more participants within a reasonable time frame from a single center would have been difficult, and hence replication of our results through larger, multicenter genetic association studies will be of interest.

## 5. Conclusions

To the best of our knowledge, this is the first study to report associations between allelic variants of the* ANKK1* rs1800497 and* LTA* rs909253 loci and risk of schizophrenia in the Egyptian population. We found that one copy of the mutant C or G allele can influence the risk of schizophrenia. Since the completion of our study, two genome-wide association studies have reported additional SNPs as risk factors for schizophrenia: the rs1006737 SNP of the* CACNA1C* gene (MIM 114205) in the European population [55], the rs548181 SNP of the* STT3A* gene (MIM 601134), the rs17603876 SNP of the* NRG1* gene (MIM 142445), and the rs3864075 SNP of the* GRM7* gene (MIM 604101) in individuals of Indo-European and Dravidian ancestry [56]. A recent study found a statistically significant excess of rare missense variants of NRXN1 (MIM 600565) and extremely rare damaging risk variants of AKAP9 (MIM 604001) in schizophrenia patients in Northwest Spain [57]. The identification of new susceptibility genes has opened new avenues for exploring the underlying disease mechanisms for schizophrenia.

## Figures and Tables

**Table 1:** Sociodemographic and clinical information of schizophrenia patients.

Parameter	Schizophrenia cases (*N* = 120)	*t* (95% confidence interval)
Ratio of women : men	48 : 72 (1 : 1.5)	
Ratio of urban : rural residence	60 : 60 (1 : 1)	
Number (%) with family history (+)^a,b^	36 (30)^c^	146.8 (0.2–0.4)^d^
Age at onset (years)	27.6 ± 10.9	27.7 (25.6–29.6)^d^
Age at examination (range, years)^e^	32.6 ± 11.27 (20–55)	31.6 (30.6–34.6)^d^
Duration of disease (years)	9.8 ± 4.7	22.7 (9.0–10.6)^d^
Number (%) with acute onset	46 (38)	
Number (%) with insidious onset	74 (62)	
PANSS score		
Total	92.8 ± 13.56	74.7 (90.3–95.3)^d^
Positive	23.1 ± 4.52	56.1 (22.3–23.9)^d^
Negative	22.7 ± 4.16	59.1 (21.9–23.5)^d^
General psychopathology	47.8 ± 7.79	67.1 (46.4–49.2)^d^
Antipsychotic medication		
Typical	84 (70%)^c^	342.8 (0.6–0.8)^d^
Atypical	36 (30%)^c^	146.8 (0.2–0.4)^d^
Typical-atypical	48 (40%)^c^	195.8 (0.3–0.5)^d^

PANSS: Positive and Negative Syndrome Scale; SD: standard deviation.

^a^Number of patients, with percentages in parentheses.

^b^Family history was considered positive (+) if there was more than one case having schizophrenia in the same family and negative (−) if the case was sporadic.

^c^ *z*-value's test.

^d^Very highly significant difference (*P*< 0.0001).

^e^Student's *t*-test. Values are mean ± standard deviation.

**(a):** 

Allele	Cases	Controls	Odds ratio	*z* (*P* value)	95% CI
	*n* (frequency)	*n* (frequency)			
rs1800497:					
T (A1)	43 (0.18)	57 (0.29)	1.8	2.9 (0.009)	1.2–2.9
C (A2)	197 (0.82)	143 (0.72)			
rs909253:					
+252A	162 (0.68)	116 (0.58)	0.7	2.1 (0.04)	0.5–1.0
+252G	78 (0.33)	84 (0.42)			

**(b):** 

Genotype	*n* (%)	*n* (%)	*χ* ^2^ (*P* value)
rs1800497:			
TT (A1A1)	0 (0.0)	12 (12.0)	15.3 (0.0005)
TC (A1A2)	43 (35.8)	33 (33.0)	
CC (A2A2)	77 (64.2)	55 (55.0)	
rs909253:			
AA	64 (53.3)	32 (32.0)	13.7 (0.001)
AG	34 (28.4)	52 (52.0)	
GG	22 (18.3)	16 (16.0)	

CI: confidence interval.

**Table 3:** Possible combined rs1800497 (T>C) and rs909253 (A>G) genotypes in schizophrenia cases and controls.

Combined genotypes	Cases, *n* (%) *N* = 120	Controls, *n* (%) *N* = 100
TT/AA	0 (0.0)	1 (0.0)
TT/AG	0 (0.0)	5 (5.0)
TT/GG	0 (0.0)	7 (7.0)
TC/AA	18 (15.0)	10 (10.0)
TC/AG	13 (10.8)	18 (18.0)
TC/GG	10 (8.3)	5 (5.0)
CC/AA	48 (40.0)	20 (20.0)
CC/AG	18 (15.0)	32 (32.0)
CC/GG	13 (10.9)	2 (2.0)

**Table 4:** Distribution of *ANKK1* rs1800497 (T>C) genotypes and their allelic frequencies in schizophrenia cases and controls from different ethnic populations.

Population^a^ (ethnicity)	Number of cases (controls)	Distribution of rs1800497 genotypes (%)^b^			C allele frequency in cases (controls)	Odds ratio (95% CI)	*P* value	Reference
		TT	TC	CC				
American (non-Hispanic white)	87 (69)	2.3 (0.0)	31.0 (14.5)	66.7 (85.5)	0.82 (0.93)	0.4 (0.2–0.8)	0.008^c^	[21]
*Egyptian* (*African Arab*)	*120* (*100*)	*0.0* (*12.0*)	*35.8* (*33.0*)	*64.2* (*55.0*)	*0.82* (*0.72*)	*1.8* (*1.2–2.9*)	*0.009* ^c^	*This study *
French (Caucasian)	144 (142)	4.2 (6.3)	30.6 (38.0)	65.3 (55.6)	0.81 (0.75)	1.5 (1.1–2.2)	0.019^c^	[38]
Brazilian (Latino)	235 (834)	12.4 (16.6)	47.6 (48.8)	40.0 (34.7)	0.64 (0.59)	1.2 (1.0–1.5)	0.062^d^	[16]
Russian (Caucasian)	311 (364)	5.8 (2.7)	33.4 (31.9)	60.8 (65.4)	0.78 (0.81)	0.8 (0.6–1.0)	0.083^d^	[42]
Indian (Indian)	212 (194)	8.0 (15.0)	44.0 (40.0)	48.0 (45.0)	0.70 (0.65)	1.3 (0.9–1.7)	0.141^d^	[41]
Chinese (East Asian)	396 (399)	16.4 (12.8)	45.7 (46.9)	37.9 (40.3)	0.61 (0.64)	0.9 (0.7–1.1)	0.209^d^	[43]
Iranian (Sri Lankan)	38 (63)	15.8 (4.8)	55.3 (61.9)	28.9 (33.3)	0.57 (0.64)	0.7 (0.4–1.3)	0.276^d^	[36]
French (Caucasian)	62 (161)	3.2 (3.1)	30.6 (28.0)	66.1 (68.9)	0.87 (0.85)	0.9 (0.5–1.5)	0.712^d^	[40]
German (Caucasian)	60 (60)	3.3 (1.7)	30.0 (30.0)	66.7 (68.3)	0.82 (0.83)	0.9 (0.5–1.7)	0.734^d^	[39]

*ANKK1*: ankyrin repeat and kinase domain containing 1; SNP: single-nucleotide polymorphism.

^a^Populations are arranged in ascending order of the *P* values.

^b^Genotype distributions of schizophrenia cases, with distributions of controls in parentheses.

^c^The distribution of the *ANKK1* rs1800497 C allele is significantly different between schizophrenia cases and controls (*P* < 0.05).

^d^The distribution of the *ANKK1* rs1800497 C allele does not differ between schizophrenia cases and controls (*P* > 0.05).
